# Trans-Ancestral Studies Fine Map the SLE-Susceptibility Locus *TNFSF4*

**DOI:** 10.1371/journal.pgen.1003554

**Published:** 2013-07-18

**Authors:** Harinder Manku, Carl D. Langefeld, Sandra G. Guerra, Talat H. Malik, Marta Alarcon-Riquelme, Juan-Manuel Anaya, Sang-Cheol Bae, Susan A. Boackle, Elizabeth E. Brown, Lindsey A. Criswell, Barry I. Freedman, Patrick M. Gaffney, Peter A. Gregersen, Joel M. Guthridge, Sang-Hoon Han, John B. Harley, Chaim O. Jacob, Judith A. James, Diane L. Kamen, Kenneth M. Kaufman, Jennifer A. Kelly, Javier Martin, Joan T. Merrill, Kathy L. Moser, Timothy B. Niewold, So-Yeon Park, Bernardo A. Pons-Estel, Amr H. Sawalha, R. Hal Scofield, Nan Shen, Anne M. Stevens, Celi Sun, Gary S. Gilkeson, Jeff C. Edberg, Robert P. Kimberly, Swapan K. Nath, Betty P. Tsao, Tim J. Vyse

**Affiliations:** 1 Department of Medical & Molecular Genetics, King's College London School of Medicine, Guy's Hospital, London, United Kingdom; 2 Wake Forest School of Medicine, Winston-Salem, North Carolina, United States of America; 3 Centre for Rheumatology & Connective Tissue Diseases, Royal Free & University College Medical School, London, United Kingdom; 4 Division of Immunology and Inflammation, Imperial College, London, United Kingdom; 5 Centro Pfizer-Universidad de Granada-Junta de Andalucía de Genómica e Investigaciones Oncológicas, Granada, Spain; 6 Center for Autoimmune Diseases Research, Universidad del Rosario, Bogota, Colombia; 7 Hospital for Rheumatic Diseases, Hanyang University, Seoul, South Korea; 8 Division of Rheumatology, University of Colorado Denver, Aurora, Colorado, United States of America; 9 Department of Epidemiology, University of Alabama at Birmingham, Birmingham, Alabama, United States of America; 10 Rosalind Russell Medical Research Center for Arthritis, University of California San Francisco, San Francisco, California, United States of America; 11 Department of Internal Medicine, Wake Forest School of Medicine, Winston-Salem, North Carolina, United States of America; 12 Arthritis and Clinical Immunology Research Program, Oklahoma Medical Research Foundation, Oklahoma City, Oklahoma, United States of America; 13 The Robert S. Boas Center for Genomics and Human Genetics, Feinstein Institute for Medical Research, North Shore LIJ Health System, Manhasset, New York, United States of America; 14 Division of Rheumatology, Cincinnati Children's Hospital Medical Centre, Cincinnati, Ohio, United States of America; 15 The Lupus Genetics Group, Department of Medicine, University of Southern California, Los Angeles, California, United States of America; 16 Department of Medicine, University of Oklahoma Healthy Sciences Center, Oklahoma City, Oklahoma, United States of America; 17 Division of Rheumatology, Medical University of South Carolina, Charleston, South Carolina, United States of America; 18 Instituto de Parasitologia y Biomedicina Lopez-Neyra, Consejo Superior de Investigaciones Cientificas, Granada, Spain; 19 Clinical Pharmacology, Oklahoma Medical Research Foundation, Oklahoma City, Oklahoma, United States of America; 20 Divisions of Rheumatology and Immunology, Mayo Clinic, Rochester, Minnesota, United States of America; 21 Sanatorio Parque, Rosario, Argentina; 22 Division of Rheumatology, Department of Internal Medicine, University of Michigan, Ann Arbor, Michigan, United States of America; 23 Shanghai Institute for Biological Sciences, Chinese Academy of Sciences, Shanghai, China; 24 Center for Immunity and Immunotherapies, Seattle Children's Research Institute, Seattle, Washington, United States of America; 25 Division of Rheumatology and Immunology, Medical University of South Carolina, Charleston, South Carolina, United States of America; 26 Division of Clinical Immunology and Rheumatology, Department of Medicine, University of Alabama at Birmingham, Birmingham, Alabama, United States of America; 27 Division of Rheumatology, Department of Medicine, David Geffen School of Medicine at UCLA, Los Angeles, California, United States of America; University of Oxford, United Kingdom

## Abstract

We previously established an 80 kb haplotype upstream of *TNFSF4* as a susceptibility locus in the autoimmune disease SLE. SLE-associated alleles at this locus are associated with inflammatory disorders, including atherosclerosis and ischaemic stroke. In Europeans, the *TNFSF4* causal variants have remained elusive due to strong linkage disequilibrium exhibited by alleles spanning the region. Using a trans-ancestral approach to fine-map the locus, utilising 17,900 SLE and control subjects including Amerindian/Hispanics (1348 cases, 717 controls), African-Americans (AA) (1529, 2048) and better powered cohorts of Europeans and East Asians, we find strong association of risk alleles in all ethnicities; the AA association replicates in African-American Gullah (152,122). The best evidence of association comes from two adjacent markers: *rs2205960-T* (*P = 1.71×10^−34^*, *OR = 1.43[1.26–1.60]*) and *rs1234317-T* (*P = 1.16×10^−28^*, *OR = 1.38[1.24–1.54]*). Inference of fine-scale recombination rates for all populations tested finds the 80 kb risk and non-risk haplotypes in all except African-Americans. In this population the decay of recombination equates to an 11 kb risk haplotype, anchored in the 5′ region proximal to *TNFSF4* and tagged by *rs2205960-T* after 1000 Genomes phase 1 (v3) imputation. Conditional regression analyses delineate the 5′ risk signal to *rs2205960-T* and the independent non-risk signal to *rs1234314-C*. Our case-only and SLE-control cohorts demonstrate robust association of *rs2205960-T* with autoantibody production. The *rs2205960-T* is predicted to form part of a decameric motif which binds NF-κBp65 with increased affinity compared to *rs2205960-G*. ChIP-seq data also indicate NF-κB interaction with the DNA sequence at this position in LCL cells. Our research suggests association of *rs2205960-T* with SLE across multiple groups and an independent non-risk signal at *rs1234314-C*. *rs2205960-T* is associated with autoantibody production and lymphopenia. Our data confirm a global signal at *TNFSF4* and a role for the expressed product at multiple stages of lymphocyte dysregulation during SLE pathogenesis. We confirm the validity of trans-ancestral mapping in a complex trait.

## Author Summary

Systemic lupus erythematosus (SLE/lupus) is a complex disease in which the body's immune cells cause inflammation in one or more systems to cause the associated morbidity. Hormones, the environment and genes are all causal contributors to SLE and over the past several years the genetic component of SLE has been firmly established. Several genes which are regulators of the immune system are associated with disease risk. We have established one of these, the tumour-necrosis family superfamily member 4 (*TNFSF4*) gene, as a lupus susceptibility gene in Northern Europeans. A major obstacle in pinpointing the marker(s) at *TNFSF4* which best explain the risk of SLE has been the strong correlation (linkage disequilibrium, LD) between adjacent markers across the *TNFSF4* region in this population. To address this, we have typed polymorphisms in several populations in addition to the European groups. The mixed ancestry of these populations gives a different LD pattern than that found in Europeans, presenting a method of pinpointing the section of the *TNFSF4* region which results in SLE susceptibility. The Non-European populations have allowed identification of a polymorphism likely to regulate expression of *TNFSF4* to increase susceptibility to SLE.

## Introduction

Tumour Necrosis Factor Superfamily (TNFS) members control wide-ranging facets of immunity when they interact with their complimentary TNF Receptors [1]. One of these, TNFSF4 (OX40L), uniquely binds its receptor, monomeric TNFRSF4 (OX40), on T lymphocytes to strongly activate NF-κB [2]. Several lines of evidence published over the last 15 years suggest the TNFSF4–TNFRSF4 interaction is required for the induction of anti-tumour immunity, allergy and autoimmunity [3]–[6] but also inhibits generation of adaptive T regulatory (TR1) cells [7]. The outcome is not limited to human disease; blockade of the TNFSF4-TNFRS4 interaction has ameliorative effects in animal models of T cell pathologies [8] including allergic and autoimmune manifestations [9]. Genetic variation at *TNFSF4* has been associated with the autoimmune disease systemic lupus erythematosus (SLE), and other inflammatory conditions including atherosclerosis and ischaemic stroke.

SLE is the prototypic multi-system autoimmune disorder. High-affinity, pathogenic IgG autoantibodies to an array of nuclear antigens are a hallmark of pathogenesis and characterise the global perturbation of the immune system in SLE. Variation in at least 25 genetic loci with modest effect sizes are thought to explain the genetic component of SLE [10]. The strong genetic basis to disease is well-established and has been strengthened by the advent of GWAS, which has corroborated the association of immunologically relevant loci with SLE [11]–[13]. We have previously shown single nucleotide polymorphisms (SNPs) in the 5′ *TNFSF4* region to be associated with lupus in European families and a case-control cohort [4]. The increased association of 5′ risk alleles with disease has been replicated in East Asian populations [14], [15], highlighting the genetic similarities at this locus in these ancestrally distinct populations. Multiple SLE risk-associated *TNFSF4* variants are also associated with systemic sclerosis [16], primary Sjögren's syndrome [17] and myocardial infarction [18], [19].

A major obstacle in the identification of disease-specific causal variants at *TNFSF4* in the European and East Asian SLE cohorts has been the strong linkage disequilibrium (*R^2^*>0.8) exhibited by genotyped *TNFSF4* alleles, which has resulted in a high frequency extended haplotype associated with risk of disease instead of delineating causal variations at the locus [4]. It is probable that migration out of Africa involved many founder effects and bottlenecks to increase haplotype length in East Asian and European populations [20]. Hispanic and African-American populations are disproportionately affected by SLE [21] and health disparities in these groups show onset at a younger age [22].

Hispanic and African-American populations have genomes which reflect recent admixture on ancient substructures [16]. Hispanic cohorts have rich diversity of source ancestry with Southern European, Amerindian and West African contribution to the inherited genome and the forced diaspora of Africans to the Americas also resulted in gene flow and two-way admixture between previously reproductively isolated West African and European ancestral populations [23]. African populations today tend to have shorter haplotypes because they usually have ancestors who have experienced more recombination events without population bottlenecks or founder effects in emigrant populations [24]. Common shorter haplotypes are often subdivisions of the larger haplotypes found in non-Africans and so can be correlated to these [25]. In admixed populations, the genetic component attributable to the West African ancestral population would equate to a faster decay of LD. The breakdown of LD is therefore greater in African-Americans, because the major component (80% or more) of their genome is West-African, compared to Hispanics who have component estimates between 4–11% [23], [26].

We infer a fine-scale map of the recombination rate and location of hotspots within each entire population and in subgroups of interest. We have used principal components (PC)-based strategies to adjust for major ancestry before performing a high-resolution association study which utilises typed and probabilistic genotypes to map the *TNFSF4* locus. By surveying common variants up to 1000 Genomes Phase 1(v3), we aim to identify common causal variation at *TNFSF4* associated with SLE. Cross-comparison of associated risk haplotypes across four populations focuses these analyses. The AA association replicates in a smaller cohort of AA-Gullah [27]. Our haplotype analyses find informative recombinants in African-Americans and Europeans to resolve genetic variants associated with SLE at this locus.

These data are used to perform six case-control association studies and a trans-ancestral mapping experiment using in excess of 17900 subjects. We attempt to define causal variation at *TNFSF4* in SLE susceptibility. In a complementary strategy we perform association analysis using *TNFSF4* alleles and lupus phenotypes. We explore the mechanism by which *TNFSF4* influences perturbation of the immune process in inflammatory disease. Finally, we interrogate risk alleles in terms of their influence on transcription factor binding using a bioinformatics approach. The research presented uses trans-ancestral mapping to inform this complex trait.

## Results

To delineate the causal variation at *TNFSF4*, we genotyped SNPs in a 200 Kb section of chromosome 1q25.1 encompassing *TNFSF4* (23.6 kb) and the 5′ region (150 kb). Population stratification bias and effects due to admixture were addressed using the approach of Namjou and colleagues [28]. We also genotyped 347 SNPs used by Halder [29] to correct for major ancestry in each population as identified by a PCA-based approach (Supplementary Figure S1). As outlined in methods, SNPs and individuals that failed quality control were filtered; pre- and post- imputation SNP counts and a description of the component sample sets is presented in **Table 1**.

**Table 1:** Population demographics and imputation reference data for SLE-control cohorts post QC filtering.

	European			East Asian			Hispanic			AA-Gullah		
	Cases	Controls	TOTAL	Cases	Controls	TOTAL	Cases	Controls	TOTAL	Cases	Controls	TOTAL
**Males**	344	1151	1495	167	225	392	119	73	192	136	593	729
**Females**	3088	2489	5577	1333	1171	2507	1229	644	1872	1541	1341	2882
**Unknown**										3	236	239
**TOTAL**	3432	3640	7072	1500	1396	2896	1348	717	2065	1680	2170	3850
**SNPS(TYPED)**	89	89	89	65	65	65	51	51	51	88	88	88
**SNPS(ALL)**	244	244	244	450	450	450	460	460	460	393	393	393
**Imputation reference 1**	1000G	1000G	1000G	1000G	1000G	1000G	1000G	1000G	1000G	1000G	1000G	1000G
**Imputation reference 2**	^OMNI-QUAD^											
	^UK-Canadian GWAS^											

Numbers after filtering for duplicates, FDRs, HWE, missingness and major ancestry. post SNPs with INFO scores <0.7 excluded, SNPS with HWE<0.01 excluded.

To directly compare genotyped SNPs we used the phased chromosomes of 1000 Genomes phase I integrated variant set v3 (March 2012, NCBI build37) (The 1000 Genomes Project Consortium) [30] and IMPUTE v2.2, together with second reference sets defined in **Table 1**. We imputed missing data and common variants (SNPs and INDELs) across the locus using MAF>1% in the imputation scaffold. As described in methods, we used a post imputation filter of HWE>0.01 and info >0.7 to include only genotyped and high quality imputed SNPs. The estimated concordance between imputed and true genotypes for the SNPs presented in this study is 0.95 for all cohorts. The final characteristics of all datasets are presented in **Table 1**.

### Bayesian inference of fine-scale map of recombination rates and hotspot densities at *TNFSF4*

**Figure 1:**
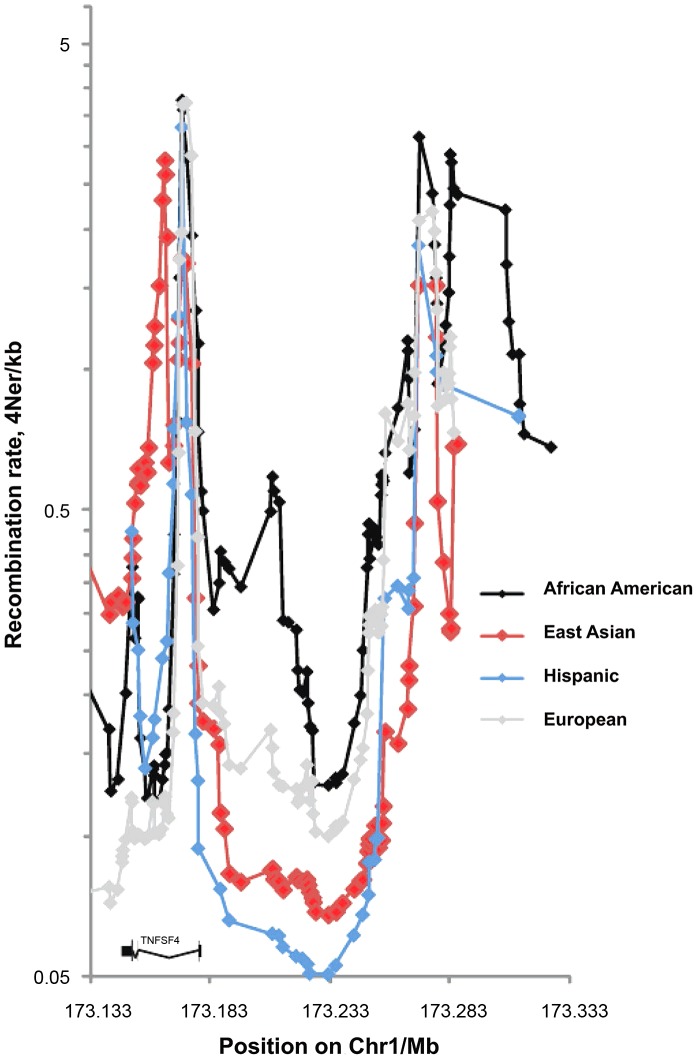
Fine scale maps of recombination rate inferred from East Asian, European, Hispanic and African-American control phased chromosomes. 1568 randomly assigned chromosomes from each group were tested using *Rhomap*, from the LDHAT2.0 package. The fine-scale map of recombination rate (4Ner/kb) was inferred across 200 kb of chromosome 1q25 encompassing *TNFSF4* gene and extended 5′ and 3′ regions.

**Figure 2:**
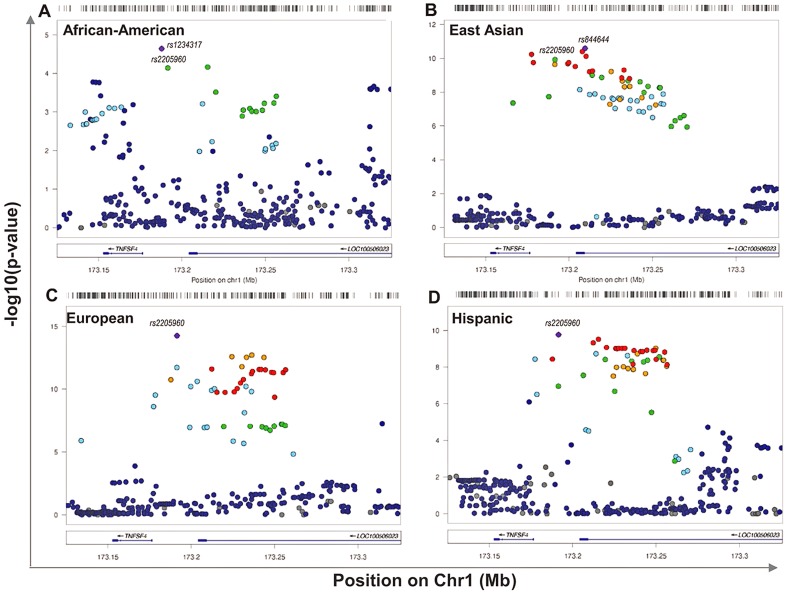
Single marker association at *TNFSF4* locus in *A*. East Asian, *B*. European, *C*. Hispanic, *D*. African-American SLE-control populations. The strength of the association (-log10uncorrected*P*) of markers across 240 kb of chromosome 1q25. This regional plot depicts *TNFSF4* association with SLE versus chromosomal position (kb) in East Asians, Europeans, Hispanics and African-American populations. The most associated variants in each group are labelled, as is the best-associated meta-analysis SNP *rs2205960*. Markers are colour-coded for their correlation coefficient (r^2^) values according to the legend.

The European sex-averaged and female-only recombination maps generated by deCODE (http://www.decode.com/addendum/), are based on 15,257 and 8,850 directly observed recombinations, respectively. These maps have a resolution effective down to 10 kb and comparing them to the HapMap 3 and 1000 Genomes population-averaged maps [30], [31], we found differences at the *TNFSF4* locus. Thus, we estimated background recombination rates in AA, East Asians, Europeans and Hispanics using a Bayesian composite-likelihood method. The inclusion of a hotspot model allowed sampling of hotspots from the Markov chain and inference of mean posterior hotspot densities from a threshold upwards of 0.25, giving a detection power of 50% and a false-discovery rate of 4% [32]. In Asians, Europeans and Hispanics the bulk of the recombination occurs in less than 5% of sequence (**Figure 1** **and** **Figure 2**). An exception to this pattern is found in the African-American cohort, with increased recombination rate and higher density and proportion of hotspots across the locus (**Figure 1**, **Figure 2**). In all populations, peak recombination is at the 5′ boundary of the *TNFSF4* gene and approximately 120 kb into the 5′ region. A difference in African-Americans is that recombination extends 30 kb from the *TNFSF4* gene boundary into the 5′ region, whilst there is negligible recombination in this section in the other populations (**Figure 1**) and this is compatible with increased complexity of the genomic region in African-Americans.

### Single marker association of 5′ *TNFSF4* SNPs with SLE

The association data presented are for markers after imputation using the 1000 Genomes phase I integrated variant set v3 (March 2012, NCBI build37) [30]. The *TNFSF4* locus is well established in SLE therefore we have presented uncorrected nominal p-values for variants. In East Asians, Europeans and Hispanics many strong associations (*P_u_ 10^−8^<10^−16^*) at *TNFSF4* are detected. Multiple susceptibility alleles in the *TNFSF4* 5′ region are overrepresented in SLE cases (**Table 2** **,** **Figure 2**). In terms of single markers, best evidence of association with disease in Europeans is observed with *rs2205960-T*, 10 kb 5′ from the *TNFSF4* gene (*P = 5.61×10^−15^*, OR = 1.34 (95%CI 1.25–1.44)). The T allele of *rs2205960* also has strongest association with Hispanic SLE (*P = 1.7×10^−10^*, OR = 1.65 (95% 1.42–1.91)). In Europeans, an additional 15 SNPs reach genome-wide significance (*P<5×10^−8^*) most of these risk alleles also reach this level of significance in the East Asian and Hispanic cohorts (**Table 2**). Several 5′ risk alleles associated with disease in East Asians, Europeans and Hispanics are also associated in African-Americans and the 5′ association replicates in a small cohort of AA-Gullah (**Supplementary Table S1**), underpinning this gene as a global SLE susceptibility gene.

**Table 2:** Single marker association results for East Asian (As), European (Eur) and Hispanic (Hisp) SLE-control cohorts.

Marker	Coordinate	F_AA/F_AB					Unadjusted p- value				Odds Ratio (95% CI)			
		AAG	As	Eur	Hisp	AA/AB	AAG	As	Eur	Hisp	AAG	As	Eur	Hisp
*rs1234314*	173.1774	0.31/0.36	0.38/0.7	0.43/0.43	0.42/0.48	C/G	3.11×10^−5^	5.84×10^−11^	2.43×10^−9^	3.67×10^−9^	0.82(0.76–0.88)	0.72(0.67–0.87)	0.83(0.78–0.87)	0.68(0.55–0.81)
*rs1234315*	173.1785	0.2/0.2	0.39/0.47	0.47/0.48	0.36/0.44	C/T	0.7	1.78×10^−10^	2.91×10^−10^	3.06×10^−7^	1.02(0.91–1.13)	0.72 (0.68–0.78)	0.82(0.76–0.88)	0.71(0.58–0.84)
*rs1234317*	173.1878	0.11/0.08	0.32/0.25	0.31/0.26	0.38/0.29	T/C	2.28×10^−5^	1.85×10^−8^	1.77×10^−11^	3.65×10^−9^	1.4(1.25–1.56)	1.37(1.26–1.48)	1.27(1.20–1.34)	1.5(1.36–1.63)
*rs2205960*	173.1915	0.07/0.05	0.32/0.25	0.27/0.22	0.37/0.27	T/G	7.20×10^−5^	1.18×10^−10^	5.61×10^−15^	1.70×10^−10^	1.48(1.2–1.67)	1.43(1.32–1.54)	1.33(1.26–1.40)	1.56(1.42–1.69)
***rs200818062***	**173.1989**	**0.1/0.07**	**0.26/0.2**	**0.27/0.23**	**0.24/0.17**	**D/I**	**2.85×10^−5^**	**5.45×10^−9^**	**2.54×10^−9^**	**3.38×10^−8^**	**1.45(1.28–1.63)**	**1.55(1.40–1.69)**	**1.26(1.18–1.34)**	**1.69(1.51–1.88)**
***rs6673550***	**173.1991**	**0.16/0.16**	**0.48/0.4**	**0.45/0.5**	**0.34/0.4**	**G/T**	**0.65**	**2.09×10^−10^**	**1.09×10^−7^**	**1.74×10^−4^**	**1.03(0.90–1.16)**	**0.71(0.66–0.78)**	**0.84(0.77–0.90)**	**0.76(0.62–0.90)**
***rs844642***	**173.2078**	**0.11/0.12**	**0.41/0.49**	**0.43/0.47**	**0.33/0.38**	**G/A**	**0.35**	**3.98×10^−11^**	**1.12×10^−7^**	**2.62×10^−5^**	**1.07(0.93–1.22)**	**0.71(0.67–0.77)**	**0.84(0.78–0.91)**	**0.74(0.61–0.88)**
*rs844644*	173.2095	0.16/0.16	0.41/0.49	0.43/0.47	0.33/0.4	A/C	0.91	2.56×10^−11^	1.04×10^−7^	3.03×10^−5^	1.00(0.88–1.13)	0.71 (0.67–0.77)	0.84(0.78–0.91)	0.75(0.62–0.89)
*rs12039904*	173.2123	0.06/0.04	0.32/0.25	0.28/0.23	0.38/0.29	A/G	6.13×10^−4^	1.38×10^−8^	2.48×10^−12^	4.71×10^−10^	1.44(1.23–1.65)	1.37(1.26–1.48)	1.29(1.22–1.36)	1.53(1.39–1.66)
*rs2795288*	173.2139	0.47/0.48	0.41/0.49	0.41/0.47	0.42/0.48	T/A	0.6	5.62×10^−10^	9.2×10^−11^	1.85×10^−9^	0.98(0.89–1.07)	0.73(0.68–0.79)	0.81(0.78–0.85)	0.67(0.55–0.80)
***rs4916315***	**173.2154**	**0.12/0.09**	**0.31/0.25**	**0.28/0.23**	**0.38/0.28**	**T/C**	**6.82×10^−5^**	**1.27×10^−8^**	**1.76×10^−10^**	**3×10^−10^**	**1.38(1.22–1.54)**	**1.37(1.26–1.48)**	**1.23(1.17–1.30)**	**1.56(1.42–1.70)**
*rs1012507*	173.2195	0.28/0.28	0.34/0.27	0.38/0.34	0.46/0.37	T/G	0.89	1.36×10^−9^	9.40×10^−8^	3.82×10^−9^	1.00(0.91–1.11)	1.39(1.29–1.50)	1.19(1.13–1.26)	1.48(1.35–1.61)
*rs35086785*	173.2202	0.13/0.11	0.31/0.25	0.28/0.23	0.39/0.29	G/T	3.05×10^−4^	2.11×10^−8^	1.79×10^−10^	8.4×10^−10^	1.29(1.15–1.43)	1.37(1.26–1.48)	1.23(1.17–1.30)	1.51(1.38–1.65)
*rs844648*	173.2243	0.29/0.28	0.42/0.36	0.33/0.28	0.44/0.35	G/A	0.34	5.17×10^−8^	2.59×10^−13^	3.07×10^−8^	1.05(0.95–1.15)	1.33(1.23–1.43)	1.28(1.22–1.35)	1.44(1.31–1.57)
*rs844649*	173.2251	0.33/0.32	0.44/0.36	0.43/0.39	0.5/0.42	C/A	0.68524	6.03×10^−10^	1.35×10^−6^	2.08×10^−7^	1.02(0.92–1.12)	1.38(1.28–1.48)	1.18(1.11–1.24)	1.41(1.28–1.54)
*rs844651*	173.2315	0.15/0.15	0.49/0.42	0.43/0.47	0.09/0.1	A/G	0.74281	1.43×10^−9^	2.08×10^−6^	0.07	0.98 (0.91–1.05)	0.73 (0.68–0.81)	0.85(0.79–0.92)	0.79(0.53–1.04)
***rs1099447***	**173.2328**	**0.47/0.47**	**0.41/0.49**	**0.42/0.47**	**0.42/0.49**	**A/T**	**0.7133**	**9.47×10^−8^**	**6.05×10^−11^**	**2.36×10^−9^**	**1.02(0.93–1.12)**	**0.73(0.68–0.79)**	**0.81(0.78–0.85)**	**0.67(0.54–0.8)**
*rs844654*	173.2361	0.12/0.15	0.25/0.31	0.23/0.28	0.29/0.38	C/A	1.28×10^−3^		5.87×10^−12^	6.91×10^−9^	0.8(0.72–0.90)	0.74(0.68–0.81)	0.78(0.74–0.83)	0.67(0.61–0.74)
*rs10489265*	173.2367	0.14/0.12	0.31/0.25	0.29/0.23	0.39/0.29	C/T	8.92×10^−4^	3.08×10^−8^	3.83×10^−12^	1.23×10^−9^	1.26(1.12–1.39)	1.36(1.25–1.47)	1.28(1.21–1.35)	1.51(1.37–1.64)
***rs34313362***	**173.2407**	**0.14/0.12**	**0.31/0.25**	**0.29/0.23**	**0.39/0.29**	**T/C**	**8.12×10^−4^**	**3.30×10^−8^**	**2.69×10^−12^**	**1.41×10^−9^**	**1.26(1.12–1.39)**	**1.36(1.25–1.47)**	**1.28(1.21–1.35)**	**1.50(1.37–1.64)**
***rs10912576***	**173.2419**	**0.14/0.12**	**0.31/0.25**	**0.29/0.23**	**0.39/0.29**	**A/G**	**9.53×10^−4^**	**1.46×10^−7^**	**2.68×10^−12^**	**1.41×10^−9^**	**1.25(1.12–1.39)**	**1.34(1.23–1.45)**	**1.28(1.21–1.35)**	**1.50(1.37–1.64)**
*rs844663*	173.2436	0.28/0.27	0.42/0.35	0.33/0.28	0.43/0.34	C/T	0.47		2.93×10^−13^	2.22×10^−8^	1.04(0.94–1.14)	1.34(1.24–1.44)	1.29(1.22–1.35)	1.45(1.32–1.58)
***rs12403570***	**173.2444**	**0.14/0.12**	**0.31/0.25**	**0.28/0.23**	**0.39/0.29**	**T/C**	**9.69×10^−4^**	**4.58×10^−9^**	**2.89×10^−12^**	**1.19×10^−9^**	**1.26(1.12–1.39)**	**1.34(1.23–1.45)**	**1.28(1.21–1.35)**	**1.51(1.38–1.64)**
*rs12049190*	173.2473	0.29/0.3	0.33/0.26	0.38/0.33	0.45/0.36	T/A	0.37	8.28×10^−8^	1.86×10^−7^	2.92×10^−6^	0.95(0.85–1.06)	1.40(1.29–1.52)	1.21(1.14–1.28)	1.41(1.27–1.56)
***rs35634597***	**173.2482**	**0.14/0.12**	**0.31/0.25**	**0.29/0.24**	**0.39/0.29**	**T/A**	**9.01×10^−4^**	**1.88×10^−8^**	**4.53×10^−12^**	**1.25×10^−9^**	**1.26(1.12–1.39)**	**1.35(1.24–1.46)**	**1.28(1.21–1.35)**	**1.51(1.37–1.64)**
***rs67638449***	**173.2493**	**0.14/0.12**	**0.31/0.25**	**0.29/0.24**	**0.39/0.3**	**T/C**	**5.93×10^−4^**	**3.16×10^−7^**	**4.91×10^−12^**	**1.2×10^−9^**	**1.27(1.13–1.40)**	**1.37(1.26–1.48)**	**1.28(1.21–1.35)**	**1.51(1.38–1.64)**
*rs12750070*	173.2498	0.19/0.17	0.33/0.25	0.39/0.34	0.46/0.36	T/C	0.01		8.77×10^−8^	9.21×10^−10^	1.17(1.05–1.29)	1.43(1.31–1.55)	1.2(1.13–1.26)	1.51(1.38–1.64)
*rs12405577*	173.2500	0.07/0.05	0.32/0.26	0.29/0.24	0.39/0.29	A/G	9×10^−3^	5.43×10^−9^	4.36×10^−10^	3.75×10^−9^	1.30(1.10–1.50)	1.34(1.23–1.45)	1.26(1.19–1.33)	1.52(1.38–1.66)
***rs6697570***	**173.2541**	**0.19/0.16**	**0.33/0.26**	**0.38/0.34**	**0.46/0.37**	**C/T**	**7.29×10^−3^**	**5.47×10^−9^**	**6.04×10^−8^**	**4.19×10^−9^**	**1.18(1.06–1.3)**	**1.377(1.27–1.49)**	**1.19(1.13–1.26)**	**1.47(1.34–1.60)**
***rs12143114***	**173.2545**	**0.2/0.17**	**0.33/0.26**	**0.38/0.34**	**0.46/0.37**	**C/T**	**8.77×10^−3^**	**4.79×10^−8^**	**6.08×10^−8^**	**4.17×10^−9^**	**1.17(1.057–1.29)**	**1.38(1.27–1.48)**	**1.197(1.14–1.26)**	**1.47(1.34–1.60)**
***rs35691278***	**173.2551**	**0.14/0.11**	**0.31/0.25**	**0.29/0.24**	**0.39/0.3**	**T/C**	**5.82×10^−4^**	**1.30×10^−8^**	**4.78×10^−12^**	**1.51×10^−9^**	**1.27(1.14–1.41)**	**1.36(1.25–1.47)**	**1.28(1.21–1.35)**	**1.50(1.37–1.63)**
***rs11802979***	**173.2563**	**0.19/0.16**	**0.32/0.25**	**0.38/0.34**	**0.46/0.37**	**T/C**	**6.54×10^−3^**	**1.30×10^−8^**	**7.82×10^−8^**	**8.94×10^−9^**	**1.18(1.06–1.30)**	**1.37(1.26–1.48)**	**1.19(1.13–1.26)**	**1.46(1.33–1.59)**
***rs10912579***	**173.2563**	**0.19/0.16**	**0.32/0.25**	**0.38/0.34**	**0.46/0.37**	**T/G**	**6.55×10^−3^**	**5.84×10^−11^**	**7.8×10^−8^**	**8.92×10^−9^**	**1.18(1.06–1.30)**	**1.37(1.26–1.48)**	**1.19(1.13–1.26)**	**1.46(1.33–1.59)**
*rs10912580*	173.2566	0.14/0.11	0.31/0.25	0.29/0.24	0.39/0.3	G/A	3.91×10^−4^	1.78×10^−10^	2.87×10^−12^	7.37×10^−9^	1.28(1.15–1.42)	1.36(1.25–1.47)	1.28(1.21–1.36)	1.48(1.348–1.61)

Variants in bold are imputed using 1000 Genomes phase 1 (v3).

In African-Americans, the best evidence for the 5′ SNP association with disease are from *rs1234317-T* (*P = 2.28×10^−5^*, OR = 1.4 (95%CI 1.25–1.56)) and *rs2205960*-T (*P = 7.2×10^−5^*, OR = 1.48 (95%CI 1.22–1.67)) and *rs1234314* –G (*P = 3.11×10^−5^*, OR = 1.22 (95%CI 1.13–1.32)). There is a trend for under-representation of the minor alleles of *rs1234314-C*, *rs1234315-C*, *rs844642-G*, *rs844644-A*, *rs2795288-T and rs844654-A in* SLE cases resulting in a flipped OR for these variants (**Table 2** **)**.

### Association of intragenic *TNFSF4* single markers with SLE

Examining the genetic association between SNPs within the *TNFSF4* gene and SLE we identify association of *rs1234313-G*, within intron1, with SLE in Asians (*P = 4.37×10^−8^*, OR = 1.38 (95%CI 1.32–1.44)), and Europeans (*P = 1.11×10^−5^*, OR = 1.15(95% CI 1.11–1.27). In both cohorts *rs1234313-G* is partitioned from other associated SNPs by a recombination hotspot at the *TNFSF4*- 5′ boundary. However, correlation coefficient *R* ^2^ values between this marker and risk-associated 5′ variants suggest strong correlation. We identify under representation of *rs10798265-A* in African-American SLE (*P = 9.24×10^−5^*, 0.84(95%CI 0.78–0.9)). There is suggestion of additional modest association signals (*P<10^−4^*) from a series of SNPs located at the *TNFSF4*- 3′UTR boundary in the same cohort.

### Imputation of typed bi-allelic indels

Imputation gave 257 common (>1% MAF) bi-allelic indels at the *TNFSF4* locus, mostly neutral. The indels were included in the same imputation analysis and subject to the same QC as the SNPs and probabilistic genotypes incorporated into our association analyses. We identify a deletion at *rs200818062* [-/G] to be associated with SLE in all groups tested. This indel is located 22.4 kb from the start site of the common transcript (Transcript 1) of *TNFSF4* and is in strong LD with (R^2^>0.8) *rs1234317* and *rs2205960*.

### Best evidence meta-analysis

We used a logistic regression model fitted with an interaction term (effect) in the R statistical package to investigate cross-study heterogeneity. P-values for individual associated SNPs were generated using a likelihood-ratio test. We found no evidence of heterogeneity for the key risk- haplotype-tagging common variants which span the locus. Our null hypothesis - that all studies were evaluating the same effect size- held true for key variants associated with risk of SLE.

We combined the association data for variants across the 5′ *TNFSF4* region in East Asians, Europeans, Hispanics and African-Americans, to more powerfully estimate the true effect size (**Table 3**). The average effect size across all datasets was computed using inverse variance weighting of each study. We find the 5′ association of *TNFSF4* with SLE greatly reinforced. *rs2205960-T*, the most associated allele in Europeans and Hispanics, (*P = 1.71×10^−34^*, *OR = 1.43[1.26–1.60]*), and *rs1234317-T* (*P = 1.16×10^−28^*, *OR = 1.38[1.24–1.54]*) have the strongest combined association with disease (**Table 3**). These adjacent markers are separated by 3 kb of chromosome 1. Allele frequencies for *rs2205960* for 1000 Genomes populations are presented in supplementary data.

**Table 3:** Best evidence meta-analysis of the association P value for *TNFSF4* SNPs.

MARKER NAME	COORDINATE (Mb)	AA/AB	FREQ	SE	WEIGHT	pValue	pValue conditioned on *rs2205960*	pValue conditioned on *rs1234314*	pValue *rs1234314*+*rs2205960*
*rs2205960*	173.1915	G/T	0.549	0.014	17096	1.71×10^−34^	1	4.12×10^−14^	1
*rs1234317*	173.1878	C/T	0.757	0.066	17096	1.16×10^−28^	0.04	2.57×10^−8^	4.57×10^−3^
*rs12039904*	173.2123	C/T	0.775	0.097	17096	2.06×10^−28^	0.12	2.31×10^−10^	0.12
*rs1234314*	173.1774	C/G	0.396	0.108	17096	6.52×10^−28^	3.81×10^−7^	1	1
*rs4916315*	173.2154	C/T	0.615	0.125	17096	8.85×10^−28^	0.01	2.64×10^−9^	0.02
*rs67638449*	173.2493	C/T	0.637	0.137	17096	9.39×10^−28^	0.10	1.25×10^−9^	0.14
*rs10912576*	173.2407	C/T	0.755	0.067	17096	1.36×10^−27^	0.08	1.67×10^−9^	0.13
*rs34313362*	173.2367	T/C	0.240	0.070	17096	1.97×10^−27^	0.07	1.95×10^−9^	0.12
*rs10912580*	173.2566	A/G	0.247	0.070	17096	2.20×10^−27^	0.09	2.63×10^−9^	0.13
*rs35691278*	173.2551	C/T	0.764	0.074	17096	2.22×10^−27^	0.06	2.62×10^−9^	0.10
*rs35634597*	173.2482	A/T	0.247	0.070	17096	5.46×10^−27^	0.06	4.48×10^−9^	0.10
*rs12046550*	173.2419	G/A	0.663	0.038	17096	5.65×10^−27^	0.05	4.82×10^−9^	0.09
*rs12403570*	173.2444	C/T	0.767	0.096	17096	5.94×10^−27^	0.05	5.14×10^−9^	0.08
*rs35086785*	173.2202	T/G	0.247	0.067	17096	1.71×10^−26^	0.02	1.16×10^−8^	0.03
*rs10489265*	173.2361	A/C	0.245	0.067	17096	3.89×10^−26^	0.04	3.49×10^−9^	0.07
*rs200818062*	173.1988	I/D	0.860	0.067	17096	4.56×10^−26^	7.09×10^−3^	1.41×10^−8^	0.01
*rs12405577*	173.2500	C/T	0.684	0.081	17096	1.24×10^−22^	0.03	2.74×10^−7^	0.03
*rs844649*	173.2243	T/C	0.396	0.042	17096	1.70×10^−22^	0.02	6.68×10^−6^	0.13
*rs844663*	173.2436	T/C	0.323	0.043	17096	1.74×10^−22^	0.01	1.83×10^−6^	0.11
*rs6697570*	173.2541	T/C	0.248	0.068	17096	1.13×10^−21^	0.24	1.05×10^−4^	0.16
*rs12143114*	173.2545	T/C	0.420	0.046	17096	1.52×10^−21^	0.25	1.34×10^−4^	0.17
*rs12750070*	173.2498	C/T	0.779	0.090	17096	2.00×10^−21^	0.16	5.21×10^−5^	0.15
*rs2795288*	173.2139	T/A	0.755	0.067	17096	2.60×10^−21^	7.74×10^−5^	4.82×10^−5^	6.61×10^−3^
*rs10912579*	173.2563	G/T	0.688	0.082	17096	3.69×10^−21^	0.14	1.86×10^−4^	0.10
*rs11802979*	173.2563	C/T	0.688	0.082	17096	3.70×10^−21^	0.13	1.86×10^−4^	0.10
*rs844654*	173.2328	T/A	0.757	0.063	17096	3.74×10^−21^	5.80×10^−5^	7.74×10^−5^	9.86×10^−3^
*rs1234315*	173.1785	T/C	0.249	0.084	17096	1.43×10^−19^	1.14×10^−4^	4.78×10^−3^	0.02
*rs1012507*	173.2195	G/T	0.661	0.044	17096	4.03×10^−17^	0.17	6.09×10^−4^	0.05
*rs844642*	173.2078	A/G	0.375	0.118	17096	4.47×10^−17^	6.6×10^−4^	3.53×10^−3^	0.04
*rs12049190*	173.2473	T/A	0.684	0.078	17096	3.33×10^−16^	0.16	2.28×10^−4^	0.08
*rs844644*	173.2095	C/A	0.672	0.044	17096	9.59×10^−16^	1.79×10^−3^	7.51×10^−3^	0.06
*rs844651*	173.2251	A/C	0.339	0.154	17096	1.27×10^−15^	0.04	7.91×10^−3^	0.12
*rs6673550*	173.1991	T/G	0.314	0.082	17096	2.65×10^−15^	3.34×10^−3^	7.29×10^−3^	0.08
*rs1099447*	173.1915	G/A	0.547	0.010	17096	3.02×10^−11^	0.03	0.06	0.37

The first three columns list SNP characteristics, the next six columns list meta-analysis results including allele frequencies (FREQ1) and two-tailed P value for the nominal SNP association, conditioned on rs2205960, rs1234314 or conditioned on both rs1234314+rs2205960.

### Conditional regression analysis of 5′ single-markers

As expected, our 5′ association data suggest pairwise LD between markers is weakest in African Americans and strongest in Asians. In order to establish whether the signals identified by our trans-ancestral fine-mapping experiment represent causal variants, independent risk factors, or if we have simply found surrogate markers strongly correlated with causal variants, we conditioned the association data from each population with the marker which represented the best evidence of association.

**Figure 3:**
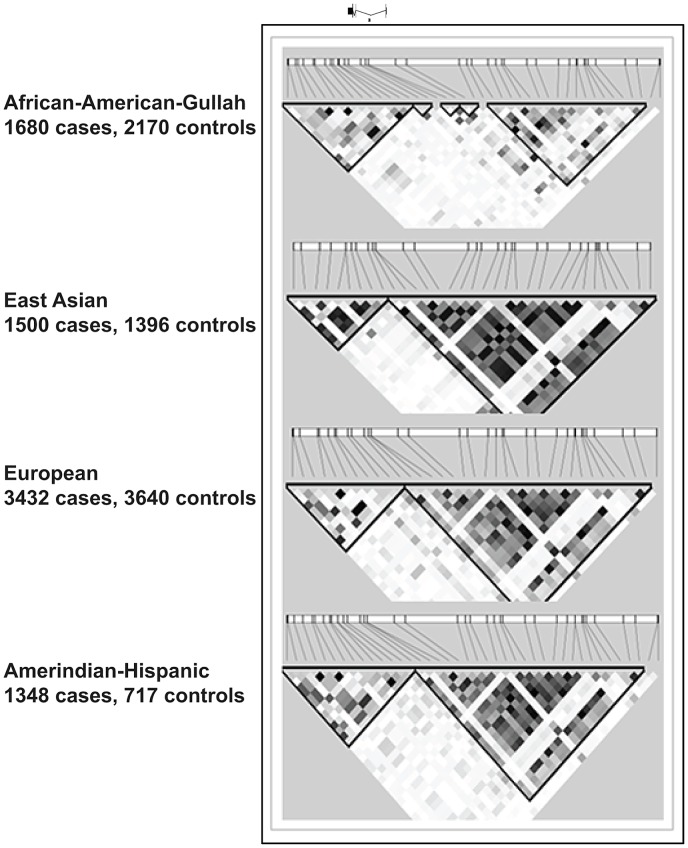
LD plots at *TNFSF4* locus in four populations. This section of chromosome 1q25.1 encompasses the *TNFSF4* gene and upstream region as defined by custom algorithm in Haploview 4.2. The measure of LD was used to depict 57 SNPs common to all cohorts, post QC and 1000 genomes imputation. The pair-wise correlations between *TNFSF4* markers is illustrated in these plots by the correlation coefficient R^2^(where r^2^ = 0 = no correlation, white; 0<R^2^<1, gradations of grey; R^2^ = 1 = complete correlation, black). The *TNFSF4* gene is positioned above the plots relative to haplotype blocks (black triangles) and grey ticks indicate SNP locations to scale.

In all populations, *rs2205960-T*, a risk-haplotype tag SNP with high p-value and effect size (**Table 3** **,** **Figure 3**) is associated with SLE; a similar trend is illustrated by the adjacent marker *rs1234317-T*. Conditioning on *rs1234317* or *rs2205960* we find the signal at *rs1234317* is lost after conditioning for r*s2205960*, *and* this is consistent for across populations (**Table 4**). If the reverse analysis is performed and we condition on the presence of *rs1234317*, there is residual association at *rs2205960* in Asians, Europeans and Hispanics (*P = 9×10^−4^_AS_*, *P<10^−4^_EU_*, *P = 0.015_Hi_*). In all apart from the AA group, conditioning on *rs1234317* or *rs2205960* leaves a residual signal at *rs1234314*. We included *rs2205960*, *rs1234317* and *rs1234314* in these analyses.

**Table 4:** Conditional regression results for 5′TNFSF4 variants in four groups.

Marker	A1	A2	Coordinate	AA+Gullah			Asian			European			Amerindian/Hispanic		
				p-value			p-value			p-value			p-value		
				rs1234314	rs1234317	rs2205960	rs1234314	rs1234317	rs2205960	rs1234314	rs1234317	rs2205960	rs1234314	rs1234317	rs2205960
*rs1234314*	*G*	*C*	*173177392*	*−1*	*0.088*	*0.103*	*−1*	*4×10^−4^*	*0.013*	*−1*	*0.017*	*0.038*	*−1*	*0.005*	*0.009*
*rs1234317*	*T*	*C*	*173187775*	*0.004*	*−1*	*0.084*	*0.190*	*−1*	*0.024*	*8.98×10^−5^*	*−1*	*0.57*	*0.007*	*−1*	*0.94*
*rs2205960*	*T*	*G*	*173191475*	*0.001*	*0.224*	*−1*	*0.008*	*9×10^−4^*	*−1*	*4.02×0^−8^*	*4.88×10^−5^*	*−1*	*3.56×10^−4^*	***0.010***	*−1*

Conditional analyses in SNPTESTv2 Case Control. Continuous covariate within a clustering framework. P values selected using additional model and a frequentist paradigm.

### Conditional analysis of meta-analysis data

We find conditioning on the presence of *rs1234317* or *rs2205960*, association of intron 1 markers tested for all groups is lost, confirming these as secondary to 5′ risk associations (**Table 2**). We also conditioned the meta-analysis association data on *rs2205960* and found residual association at *rs1234314* (*P = 3.81×10^−7^*), located at the *TNFSF4*-5′ boundary. The reverse analysis found increased residual association at *rs2205960* (*P = 4.12×10^−14^*). These data suggest two independent signals with increased association and effect at *rs2205960* compared to *rs1234314* in SLE. Conditioning the meta-data on both *rs1234314* and *rs2205960* removed association at *TNFSF4* (**Table 3**).

### Modelling the pattern of inheritance in independent cohorts

In genotype-based analyses, the models that best fits the 5′ association of *TNFSF4* with SLE are the additive/dominant models.

### Haplotype bifurcation of *TNFSF4* risk and non-risk haplotypes

**Figure 4:**
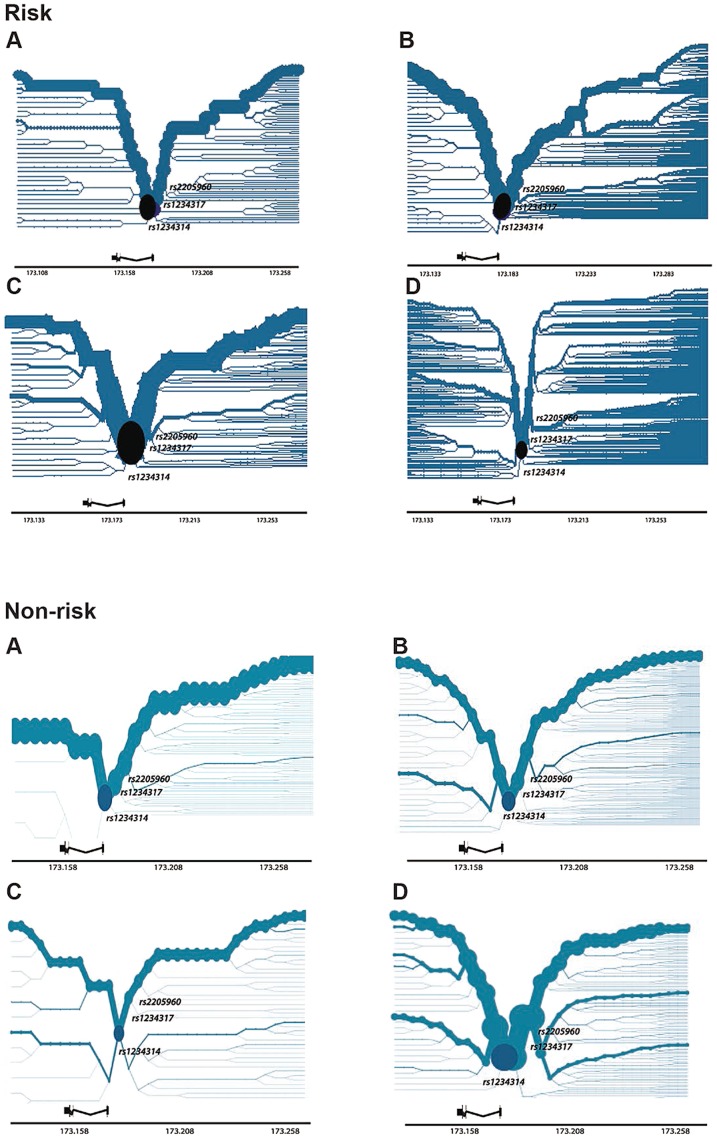
Haplotype Bifurcation Diagrams of *TNFSF4_risk_* and *TNFSF4_non-_*_**risk**_ for Four Populations. Plots are constructed using phased haplotypes for a. *East Asians*, b. *Europeans*, c. *Hispanics* and d. *African-Americans* and illustrate breakdown of LD with increasing distances from a core proximal *TNFSF4* SNP and are approximately to scale. The core is located at the *TNFSF4* gene-5′ boundary (black circle) and is selected as the most proximal 5′ marker, *rs1234314*, in each population. Gene location is depicted to scale; we have additionally labelled each plot to show the location of *rs1234317* and *rs2205960*, the best-associated markers from the meta-analysis.

Haplotypes significantly associated with risk of disease were identified for each population. To better visualise the breakdown of LD of associated haplotypes, we constructed bifurcation diagrams from phased genotypes for each cohort tested (**Figure 4** **, risk**). The plots illustrate the breakdown of linkage disequilibrium (LD) at increasing distances in both directions from *rs1234314*, the most proximal genotyped SNP located at the *TNFSF4* gene-5′ boundary and which is used as the core variant in the figure (labelled, circular core from which haplotype branches). The location of *rs1234317* and *rs2205960*, best-associated in the meta-analysis, are also marked onto the diagram. The thickness of the line in each plot corresponds to the number of samples with the haplotype, branches indicate breakdown of LD. For the risk haplotype, the lines are most robust in East Asians (**Figure 4** **, risk**), followed by Hispanics and Europeans, and least robust in African-Americans. We find branch junctions depicting breakdown of LD on the risk haplotype to be coincident with the section of the *TNFSF4* locus encompassing *rs1234317* and *rs2205960*.

The non-risk haplotype retains its thickness with distance from the core in the AA group, indicating long-range homozygosity (**Figure 4** **, non-risk**). Contrasting the recombination rate in risk and non-risk haplotype homozygotes finds increased recombination in the risk individuals (**Supplementary Figure S3**), supporting these bifurcation data.

### Conservation of *TNFSF4* haplotype structure at the TNFSF4 locus in ancestrally diverse populations

Significantly associated haplotypes are found in each population (**Table 5**). Low recombination and similar location of hotspots at the *TNFSF4-*5′ boundary in East Asians, Europeans, and Hispanics allow for the construction of near-identical haplotype blocks including risk and non-risk haplotypes (designated *TNFSF4_OR>1_* and *TNFSF4_OR<1_*, respectively)(**Figure 3**) which extend approx. 80 kb into the *TNFSF4* 5′ region. Multiple associated risk alleles uniquely tag *TNFSF4_OR>1_*, the risk haplotype, which is overrepresented in SLE individuals in each population, whilst *TNFSF4_OR<1_* is the most frequent haplotype for all cohorts tested but underrepresented in SLE individuals.

**Table 5:** *TNFSF4* haplotype association in four SLE-control populations.

	Start, bp	End, bp	Size/kb	Freq	p-value	OR
*RISK*, *TNFSF4_OR>1_*						
AA	173187775	173198892	11.1	0.05	8.11×10^−5^	1.56
AS	173177392	173256550	79.2	0.26	3.14×10^−7^	1.34
EUR	173175832	173256550	80.7	0.21	4×10^−13^	1.35
HIS	173177392	173256550	79.2	0.3	4.2×10^−9^	1.57
*NON-RISK*, *TNFSF4_OR<1_*						
AA	173175832	173187775	11.9	0.48	2.2×10^−5^	0.82
AS	173177392	173256550	79.2	0.52	9.35×10^−9^	0.74
EUR	173175832	173256550	80.7	0.39	1.8×10^−7^	0.8
HIS	173177392	173256550	79.2	0.31	7.1×10^−6^	0.76
*NEUTRAL*,*TNFSF4_OR∼1_*						
AA	173187775	173198892	11.1	0.03	0.1	1.05
EUR	173175832	173256550	80.7	0.04	0.44	1.03

The risk haplotype found in East Asians, Europeans and Hispanics is fragmented in the African-American cohort; the most associated risk haplotype is 11 kb (*P = 2.12×10^−5^*, OR = 1.52). This haplotype block extends from *rs1234317* to the bi-allelic indel *rs200818062*. Only one allele uniquely tags this haplotype, *rs2205960-T*, the associated alleles of *rs1234317-T* and *rs200818062-* are also found on a completely neutral haplotype. This haplotype block is separated from the adjacent distal block by *R^2^ = 0.33*. Haplotype association data for *TNFSF4_OR>1_* and *TNFSF4_OR<1_* are presented in **Table 5**.

In Asians, Europeans and Hispanics, the non-risk haplotype is tagged by *rs1234314-C*, *rs1234315-C*, *rs844642-G*, *rs844644-A*, *rs2795288-T and rs844654-A*. These variants have a flipped OR (**Table 2**) and there is residual signal at these variants after conditioning on risk-associated variants. In African-Americans, there is a signal at *rs1234314-C*. Conditioning our meta-analysis data on *rs2205960-T* there is residual association at each of these variants and the OR for the minor allele is flipped. The best-associated variant after conditioning on the risk signal is *rs1234314-C*. This variant is associated in all groups tested and resides at the *TNFSF4*-5′ boundary. Conditioning on *rs1234314* and *rs2205960* removes association at *TNFSF4*.

### Conditional regression analysis of haplotypes

In all groups, we conditioned upon the presence of *TNFSF4*_OR>1_ and found residual association of *TNFSF4_OR<1_*. Reversing the analysis by conditioning on presence of *TNFSF4_OR<1_* also finds residual association of *TNFSF4*_OR>1_. These analyses demonstrate that the observed signals in the *TNFSF4* promoter region independently confer risk and protection against SLE.

### Informative neutral haplotypes provide support for causal SNPs identified by conditional analysis

We found haplotypes in the European and AA cohorts which are tagged by the risk allele *rs1234317-T* but the non-risk allele *rs2205960-G* and not associated with disease. In African-Americans, the alleles of *rs1234317-T* and *rs200818062-* are found on a neutral haplotype, not associated with SLE. This haplotype block is separated from the adjacent distal block by a correlation coefficient value R^2^ = 0.33. These data support our conditional regression data which indicate *rs2205960-T* as the driver of the risk association.

### Subphenotype analyses

Given *TNFSF4* surface expression on a range of cell types which control immune functionality, one might expect *TNFSF4* alleles to be associated with disease manifestations of SLE. Median (IQR) age at diagnosis, autoantibody production and renal disease were examined within SLE cases and against controls in each ancestral group. American College of Rheumatology (ACR) classification criteria [33] were additionally examined in East Asians, Europeans and Hispanics. Phenotypic subsets of SLE cases are less heterogenous than SLE per se and so may enrich for risk variants with increased effect size or prove informative for causal mechanism. Clinical characteristics of SLE individuals sorted by population are presented with case-only and phenotype-control association results (Supplementary Table S2).

### Association of *TNFSF4* markers with autoantibody production

Case-only analysis reveals association of *TNFSF4* risk variants with autoantibody production in African-American, European and Hispanic SLE cohorts: Evidence of association of *rs2205960-T* with Anti-Sm autoantibodies in African-American cases (*P = 5.1×10^−3^*, OR = 1.57(95% CI 1.14–2.16) is reinforced by testing this variant against controls (*P = 6.67×10^−7^*, OR = 1.91(1.47–2.47)). We find this marker also segregates with Anti-Sm autoantibodies in European case-only and phenotype-control analyses. In Europeans the adjacent variant *rs1234317-T* is associated with Anti-Ro autoantibodies (*P = 9.5×10^−4^*, OR = 1.31(95% CI 1.12–1.54) and this is reinforced against controls (*P = 9.5×10^−8^*, OR = 1.52 (1.3–1.76)). In African-Americans analyses of 5′ variants against controls improves the significance of risk-haplotype-tagging variants with Anti-dsDNA autoantibodies (*rs1234317-T*, *P_u_ = 5.36×10^−6^*, OR = 1.68(95%CI 1.34–2.1.)) We find a transancestral trend of underrepresentation of *TNFSF4* intron 1 alleles with autoantibody production (Hispanic *P = 1.7×10^−4^*, OR = 0.52(95% CI 0.36–0.73), European *P = 2.5×10^−3^*, OR = 0.81(0.7–0.93) and East Asian *P = 3.6×10^−2^*, OR = 0.7 (95% CI 0.5–0.98)). Conditional regression analysis of the best-associated marker in each population removes all evidence of association.

### Association of *TNFSF4* markers with age of onset

Examination within cases also reveals association of distal 5′ *TNFSF4* alleles with age of onset (IQR) across all cohorts examined apart from East Asians (Supplementary Table S2). We classified the first and last quartile of age of onset into early and late onset in the analysis. Underrepresentation of distal 5′ *TNFSF4* alleles in lupus individuals with early age of onset is found in AA (*P = 9×10^−4^*, OR = 0.69 (95% CI 0.56–0.86)), European (*P = 1.43×10^−3^*, OR = 0.78(0.68–0.91)) and Hispanic (*P = 1.43×10^−3^*, OR = 0.57(95% CI 0.41–0.81)) populations. Inverse square meta-analysis finds the marker with best evidence of association with this phenotype (*rs844654-A*, *P = 8.7×10^−6^*, Z score 4.45), 60 Kb from the *TNFSF4* gene-5′ boundary.

### Identification of 5′ end of putative *TNFSF4* transcripts

**Figure 5:**
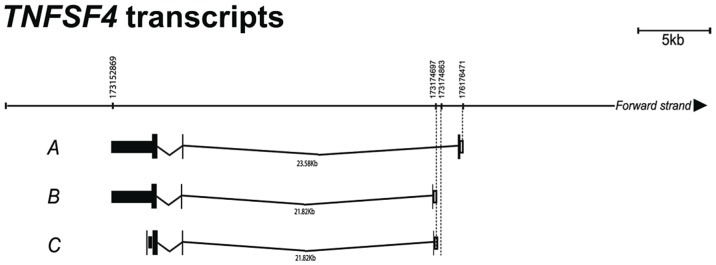
Confirmation of *TNFSF4* start site and splice variants. 5′ RACE analysis was used to map the *TNFSF4* transcription start site. Three out of four putative splice variants modelled by the Aceview tool in the UCSC genome browser were validated in a European individual. Splice variants a. and b. are protein coding, whilst variant c. is transcribed but not translated.

To gain further insight into the transcriptional regulation of the *TNFSF4* gene we analysed the 5′ ends of four putative *TNFSF4* transcripts from the activated B lymphocytes of a European individual. We evaluated the mRNA predictions for *TNFSF4* because the Gencode mRNA predictor annotates three *TNFSF4* splice variants, whilst Aceview, which has increased sensitivity for the cDNA-supported transcriptome, annotates four mRNA splice variants [34]. To position our association data accurately against the *TNFSF4* gene, we generated the 5′ ends of transcripts by 5′ RACE-PCR and found multiple transcripts which differ in their first exon usage (**Figure 5**) including a transcript for what is likely to be a soluble form of *TNFSF4* (**Figure 5**), this transcript maps identically to a transcript found in the Ensembl and UCSC genome browsers, but is yet to be found translated. We have anchored our association data to the most abundant transcript (Transcript A, **Figure 5**) sequenced.

### Association of *TNFSF4* variants with expression in LCLs

Expression profiling of common *TNFSF4* variants was carried out in a *cis* eQTL study in LCL samples from 777 female TwinsUK participants [35]. Association of RNA expression with >2×10^6^ SNPs was tested by two-step mixed model–based score test [35]. To characterize likely independent regulatory effects, the identified *cis* eQTLs were mapped to recombination hotspot intervals. For each gene, the most significant SNP per hotspot interval was selected, and LD filtering performed. The top-*cis*-eQTL in the LD bin, for the probe located at *TNFSF4* (ILMN_2089875), was *rs2205960* (*P = 3.75×10^−4^*).

### Bioinformatic analysis

**Figure 6:**
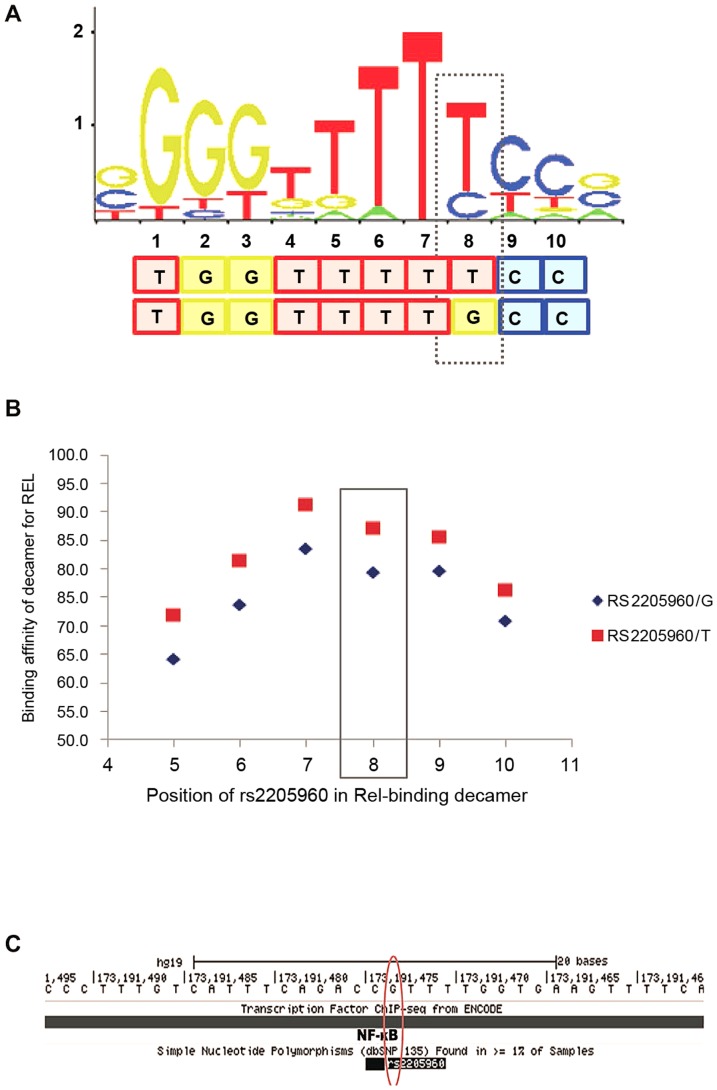
SLE-associated *rs2205960* predicted to be part of a decameric motif for NF-κB p65 (RELA). *A*. Degeneracy within the core 10-base motif is illustrated at all positions apart from position 7 which is non-degenerate by the stacked letters at each position. The relative height of each letter is proportional to its over-enrichment in the motif. A dashed line is boxed around *rs2205960-T*, this SLE-associated allele is predicted to form the 8^th^ nucleotide in the motif. Predictions were made using the non-degenerate set of matrix profiles in the Jaspar Core database. *B*. Altering the *rs2205960* allele from *-T* to *-G* decreases the binding affinity for NF-κB p65 by over 10%. *C*. Binding of NF-κB at *rs2205960*, suggested by genome-wide ChIP-seq ENCODE data. Profiles were generated for lymphoblatoid cell lines and stored in the UCSC genome database.

We examined the interaction of individual transcription factors (TFs) and other proteins with the DNA sequence at *rs2205960*. A decameric DNA sequence including the *rs2205960* variant at the 8^th^ position was predicted to bind to the NF-κB p65 protein (RELA) with high confidence. We investigated changing *rs2205960 allele*, from the minor (T) to major (G) allele and its impact on binding affinity of the motif for the target protein, p65. Using SELEX binding data and position weight matrix (PWM) profiles stored in the Jaspar core database [36], we found the DNA sequence with *rs2205960-T* at the 8^th^ nucleotide position had a binding affinity of approximately 90% for NF-κB p65 (**Figure 6**). Altering the allele to *rs2205960-G* decreased the binding affinity for NF-κB p65 by over 10%, but highlighted degeneracy of the motif (**Figure 6b**). Binding of NF-κB at *rs2205960* has been confirmed by genomewide ChIP-seq experiments in EBV - B cell lines as part of the ENCODE project (**Figure 6c**) [37]. These ChIP-seq data indicate that signal intensity for NF-κB at *rs2205960* in a heterozygous (G/T) cell-line (GM12878) is double that for a non-risk homozygote (G/G) cell line (GM06990).

We further examined the sequence encompassing *rs1234314* for transcription factor binding. According to our conditional analysis, *rs1234314* is the best-associated variant after conditioning on the risk-association. Furthermore this variant tags the non-risk haplotype. Scanning the data held in the Ensembl genome browser revealed *rs1234314* to be part of a 400 bp segment which has repressed/low activity in LCL cells but with no such activity in a T cell line. The UCSC genome browser predicts *rs1234314* to be located within a region associated with the H3K27Ac chromatin signature which is associated with active enhancers. Interrogating the sequence at *rs1234314* with PWM binding data in the Jaspar core database gave no clear pattern of binding of either allele to the motif of a regulatory element.

Examining the sequence with *rs1234317-T* against PWM binding data stored in the Jaspar Core database finds it completes a TATATT-binding motif and this motif is disrupted in the presence of *rs1234317-C*. The ENCODE project does not highlight binding of a TBP protein at this variant. Genome-wide ChIP-seq data from the ENCODE project has data for LCLs which carry the T allele of *rs1234317*. For LCLs carrying the risk (T) allele, there are currently no regulatory features annotated at this position.

## Discussion

We present a trans-ancestral fine-mapping association study of *TNFSF4* in SLE. We have genotyped haplotype-tagging and proxy variants and major ancestry informative markers in 6 populations, including admixed groups, across 200 kb of 1q25 encompassing the *TNFSF4* gene, and 5′ and 3′ regions. We also present a fine mapping association analysis of *TNFSF4* SNPs in African-American SLE. Association testing of *TNFSF4* variants revealed strong association of 5′ variants with disease in all cohorts (Tables 2–5) establishing it as a global lupus susceptibility gene. Resolution of the association signal was aided by increased recombination in the AA group (Figure 1), and by increased power from the large numbers in our European cohort. Maximal power was achieved testing with a genetic model concordant with the major underlying mode of inheritance of the 5′ *TNFSF4* region in SLE, which is additive. Our study would suggest trans-ancestral mapping as a useful tool where linkage disequilibrium is an obstacle.

Testing most of the common polymorphisms at the locus allowed identification of additional candidate variants that might underlie association at *TNFSF4*. As expected, most high-frequency SNP probabilistic genotypes included in this study are present in dbSNP; especially in the *TNFSF4* gene itself. Prior to QC filtering, the African-American population contributed the largest number of probabilistic genotypes at SNP loci. Although our ability to impute bi-allelic indels accurately from the 1000 Genomes Project resource is limited by FDR, it still increased power to detect association signals at a majority of common small indel sites accurately. In excess of 50% of the indels in the imputation scaffold were novel in all groups. We mention the bi-allelic deletion, *rs200818062*, which is in LD with our best-associated variant, *rs2205960*, and which is associated with SLE in all cohorts tested. Our AA and European data suggest this risk-associated deletion is found on a neutral haplotype which is not associated with disease. After QC filtering of imputed variants in these populations, our data suggests no new imputed variant better explained the risk signal than the typed SNP *rs2205960-T*.

A key limitation of this study is *TNFSF4* imputation may have missed common variations located in the distal 5′ *TNFSF4* region which could be causal. Accurate characterisation of variants remains challenging in low-complexity regions including the LINE element found in the distal 5′ section of this locus. As a result, variants in this region are systematically underrepresented in genetic association studies. Furthermore, an association signal may reside in the fraction of SNPs which have a lower imputation performance and were omitted using our info threshold of 0.7. This fraction is likely to include rare variants which are too infrequent to be imputed with confidence but which might have a large effect on risk. However, our data suggest the true causal variants are likely to be common (>5% frequency) and located in the proximal section of the 5′ region. The standard error of the beta coefficients for most imputed variants included in later analyses reflect high imputation certainty.

Mapping the alleles uniquely tagging the risk haplotype in each cohort has established the boundaries of risk and non-risk haplotypes in East Asians, Hispanics, and African-Americans and validated the haplotype boundaries previously defined in Europeans [4]. We avoided spurious associations through poor matching of cases with controls by the removal of outlying individuals (Supplementary Figures S1 and S2) and tested the association of risk alleles across all groups in this study.

Comparing recombination patterns in African-American individuals homozygous for the risk and non-risk haplotypes finds increased recombination in the risk-haplotype. Our results provide evidence for global association of *rs2205960-T* with SLE and assessment of the contribution of *rs2205960-T* to disease risk by conditional regression suggests that this allele drives the 5′ *TNFSF4* association in African-Americans, Europeans and Hispanics. Increased decay of 5′ LD at *TNFSF4* in AAs anchor the associated haplotype to the proximal 5′ region of *TNFSF4*. Examining the LD structure at *TNFSF4* in African-Americans and Europeans validates our association data: Neutral haplotypes in these populations, recombinant between *rs1234317*, *rs2205960* and r*s200818062-*, support our conditional regression results. Association testing within the Anti-Smith autoantibody-positive AA lupus subgroup strengthens the association P value and effect size of *rs2205960-T* and this trend replicates in Europeans (Table S2).

Curated and non-redundant profiles of SELEX binding experiments, stored in the JASPAR core database [36], suggest *rs2205960-T* would form the 8^th^ nucleotide of a decameric motif with high binding affinity for NF-κB p65 (Figure 6). Altering the 8^th^ nucleotide of the decamer to *rs2205960-G* reduces the binding affinity of this sequence for this NF-κB protein by approximately 10%, according to these data. ChIP-seq data generated for two Hapmap lymphoblastoid cell lines confirm binding of NF-κB at this location. ENCODE ChIP-seq data also suggest binding of the transcription factors BCL11a, MEF2a and BATF at *rs2205960*, albeit with lower signal intensity compared to NF-κB. These data suggest the genomic region encompassing *rs2205960-T* to have strong regulatory potential.

These data were generated for the ENCODE project [37], and establish that a positive signal for NF-κB binding is found at *rs2205960* but not *rs1234317*. A signal is found in both cell lines and there is increased signal intensity in the risk/non-risk heterozygote compared to the non-risk homozygote cell line. These data suggest a mechanism by which *rs2205960-T* could increase gene expression, which may underlie the SLE risk.

Our data suggest a putative role for *TNFSF4* in autoantibody generation, further clarifying the role of this gene in lupus pathogenesis. Correlation of *rs1234317-T* with the presence of anti-Ro autoantibodies in European cases is strengthened against controls. The Genomatix SNP analysis web tool predicts *rs1234317-T* to destroy the DNA binding site for the transcriptional repressor E4BP4, a transcription factor with a role in the survival of early B cell progenitors [38]. The DNA sequence encompassing either the C or T allele of *rs1234317* was investigated for binding to this transcription factor using the curated set of binding profiles stored in the Jaspar core database. We could not confirm binding of the sequence with either allele to the E4BP4 repressor with these data. However, the T-allele of *rs1234317* completes a TATATT consensus sequence for the TATA-Binding Protein (TBP). External sources of regulatory data stored in Ensembl and UCSC do not validate the binding of TBP or other members of the transcription initiation complex. The genomewide ChIP-seq data from the ENCODE project has data for LCLs which carry the T allele of *rs1234317* associated with SLE risk. We would expect enrichment for TFs such as TBP, or marks of open chromatin, but there are currently no data for LCLs carrying the risk (T) allele. However binding of this factor is associated with transcription initiation and so this variant merits further investigation in Rho- autoantibody-positive subsets of SLE individuals.

Association of *rs2205950-T* with African-American lupus concurs with data published previously by our group establishing a 5′ *TNFSF4* association with SLE in Northern Europeans [4]. The risk-associated variants *rs2205960-T* and *rs1234317-T* are strongly associated in the Minnesota cohort consistent with our results in four racial groups. In this previous study LD was a major obstacle in delineation of causal variation. Crucially we find association testing using a very large number of Europeans and the admixed AA group allow delineation of the signal through conditional analyses and the presence of neutral recombinant haplotypes. The African-American data presented does not validate data presented by Delgado-Vega and colleagues [39], suggesting *rs12039904-T* and *rs1234317-T* to explain the entire haplotypic effect at *TNFSF4* with SLE. A possible explanation for the modest association of *rs12039904-T* in our African-American cohort is that it is monomorphic in West African populations such as the Yoruba from Ibadan, Nigeria. Our data find *rs12039904-T* a borderline rare allele in African-Americans and we find nominal allelic association of *rs12039904-T* with disease, conditional regression analyses of *rs2205960* results in absence of an association signal at *rs12039904* in all groups.

Sanchez and colleagues use *TNFSF4 rs2205960* and single markers at 15 other lupus susceptibility loci to illustrate correlation of Amerindian ancestry with increased frequency of lupus risk alleles [40]. Delineation of *rs2205960-T* in the context of LD with adjacent markers isn't the aim of the Sanchez study, as a single SNP is typed at each locus. They find aggregation of deleterious alleles in Amerindian SLE individuals which are complemented by the increased effect sizes we find for associated *TNFSF4* variants in Amerindians and Hispanics in this study.

In Asians, Europeans and Hispanics, the non-risk haplotype is tagged by *rs1234314-C*, *rs1234315-C*, *rs844642-G*, *rs844644-A*, *rs2795288-T and rs844654-A*. In African-Americans, there is a signal at *rs1234314-C and a* weaker signal at *rs844654-A*. Conditioning our meta-analysis data on *rs2205960-T*, the variant which is best-associated with risk in this study, there is residual association at each of these variants and the OR for the minor allele is flipped. The best-associated variant after conditioning on the risk signal is *rs1234314-C*. This variant is associated in all groups tested and resides at the *TNFSF4*-5′ boundary. Conditioning on *rs1234314* and *rs2205960* removes association at *TNFSF4*.

In summary, the data presented establish *TNFSF4* as a global susceptibility gene in SLE. We have replicated and refined the 5′ association with disease and anchored risk and non-risk signals to the proximal *TNFSF4* promoter region through our efforts in African-Americans, and in Europeans by virtue of increased power in this large cohort. Recombination at the locus in African-Americans, and the conditional regression strategies employed, focus the 5′ *TNFSF4* association with disease to *rs2205960-T*. This variant uniquely tags the risk- haplotype in African-Americans and is strongly associated with disease in all groups tested. We find this marker segregates with autoantibody subsets in African-Americans, European and Amerindian/Hispanic groups. Furthermore, ChIP-Seq and bioinformatic data suggest that *rs2205960-T* sits within DNA that binds NF-κBp65 (RelA). This suggests that the risk allele would convey greater responsiveness of *TNFSF4* expression to an NK-κB stimulus. Collectively, these data confirm cross-ancestral *TNFSF4* association with SLE and suggest trans-ancestral mapping a useful strategy in complex traits.

## Materials and Methods

### Subjects

European samples held as part of the UK SLE and control collection held at Kings College London (KCL) were approved by 06/MRE02/009; additional AA samples from the CASSLE group were held at the University of Alabama at Birmingham (UAB) and approved by the UAB IRB. This study included over 17,900 SLE and control individuals of self-reported European, African-American (AA), AA-Gullah, East Asian and Hispanic/Amerindian ancestry. All cases fulfilled four or more of the 1997 ACR revised criteria for the classification of SLE and provided written informed consent. Samples were collected from multiple sites with Institutional Review Board (IRB) permission and processed at the Oklahoma Medical Research Foundation (OMRF) under guidance from the OMRF IRB.

### Phenotypes

Clinical data on SLE manifestations in all subjects were obtained from medical record review performed at individual institutions, collected and processed at the OMRF, with additional phenotypic information from KCL, MUSC and UAB.

### Genotyping and quality control

Genotyping was performed in two independent experiments on the Illumina iSelect platform at OMRF for combinations of haplotype tag SNPs and proxy variants capturing all common haplotypes, this meant we did not type all markers in all groups, marker selection was dictated by *TNFSF4* locus architecture and included SNPs found to be associated in our previous European association study [4] and Hapmap phase 3 populations [31]. In all, 125 different SNPs in a 200 Kb region (chromosome 1, 171,400,000–171,600,000 NCBI build 36.3) encompassing the *TNFSF4* gene and 5′ region were genotyped.

Population stratification bias and effects due to admixture were addressed by genotyping 347 genome-wide SNPs as used by Halder and colleagues [29] to correct for major ancestry. 20 Additional 1q25-specific ancestry markers were genotyped to correct for two-way admixture between Europeans and AAs. Within each population, Eigenstrat was used for principal components (PC) analysis and global ancestry estimates were additionally inferred by a combined Bayesian and sampling-theory approach (Admixmap). We spiked the African-American population with Yoruba, Tuscan and Northern/Western European Hapmap III individuals to cross-compare two-way admixed AAs with their founder populations (Supplementary Figure S1).

Markers with less than 90% genotyping efficiency were excluded from the analysis. Hardy-Weinberg Equilibrium (HWE) was assessed in control samples of each cohort. We included markers which deviated up to a *P>0.01* threshold for HWE. We also included markers which had an acceptable HWE p-value in three of the four cohorts, if associated with SLE in multiple populations. Following filtering for duplicates, first-degree relatives, HWE, missingness and major ancestry, the non-imputed dataset comprised 111 *TNFSF4* SNPs and 294 AIMs and 17900 samples prior to imputation (Table 1).

### Imputation methods

Imputation of the genomic region from 173,112,930 to 173,349,886 (NCBI build 37) on chromosome 1q25.1 was performed using IMPUTE2.2 and the phased haplotypes from the 1000Genomes phase-1 integrated_v3 dataset [31]. Genotypes from our UK-Canadian GWAS (unpublished) were used as a second reference for the imputation of the European cohort (Table 1). Our aim was to fill missing gaps in the genotyping data and impute common markers (>1% MAF) missing between datasets to examine association at *TNFSF4* and to better inform the structure of common haplotypes across the populations. We estimated concordance between imputed and true genotypes and Imputed SNPs were included in downstream analysis if SNP info scores exceeded 0.7 and a HWE>0.01. These criteria successfully filtered out all but the best-imputed SNPs.

### Inference of population-specific recombination maps

We used FASTPHASE to phase 6272 unrelated control chromosomes (1568 from each population), randomly chosen after QC filtering. *Rhomap* from the LDhat2.0 package [32] was used to estimate population scale recombination rates in the presence of hotspots using pre-computed maximum likelihood tables in the analysis. Using the approach of Auton and colleagues, *Rhomap* was run for a total of 1,100,000 iterations including a burn-in of 100,000 iterations, the chain was sampled every 100 iterations after the burn-in. Each simulation incorporated 196 chromosomes meaning a total of 8 simulations were completed per group and the mean average recombination calculated between each pair of markers at the *TNFSF4* locus. Simulations were executed in their entirety on 3 separate occasions to ensure there were no irregularities. The data did not change if we increased the parameters used. These analyses were then extended to infer recombination in phased chromosomes from African-American risk and non-risk homozygote individuals (Supplementary Figure S3).

### Single marker association analyses

After QC filtering, single marker association and conditional data were generated using a case-control format and the continuous covariate function in SNPtestv2 under the additive model. We used a frequentist statistical paradigm and a probabilistic method for treating genotype uncertainty. Odds ratios (OR) with 95% confidence intervals (95% CI) were calculated using the beta statistic and 95% confidence intervals the SE. Data are represented as nominal uncorrected p-values.

### Meta-analysis

We used a logistic regression model fitted with an interaction term (effect) in the R statistical package to investigate cross-study heterogeneity. P-values for individual associated SNPs were generated using the likelihood-ratio test. We found no evidence of cross-study heterogeneity for the key haplotype-tagging common variants which span the locus. These were *rs1234314*, *rs1234317*, *rs2205960*, *rs12039904*, and *rs10912580*. We have presented the results of a fixed-effects meta-.results for East Asians, Europeans and Hispanics and African-Americans to more powerfully estimate the true effect size (Table 3). The effect size across all datasets was computed using inverse variance weighting of each study.

### Bifurcation

By using the Long Range Haplotype (LRH) test to look for common alleles with long-range linkage disequilibrium (LD), we were able to represent the breakdown of the risk haplotype, *TNFSF4*_risk_. *TNFSF4*_risk_ was anchored by *rs1234314* in all groups, a marker conveniently positioned at the boundary of the *TNFSF4* gene and 5′ region. Haplotype bifurcation diagrams were generated in the program Sweep. Two SNPs which show best evidence of association after meta-analysis, *rs1234317* and *rs2205960*, are marked on the scale of each bifurcation plot.

### Haplotype association and conditional regression

Haplotypes in the *TNFSF4* gene and 5′ region were generated using Haploview 4.2 using the custom algorithm, based on the R^2^ measure of linkage disequilibrium (LD). Markers and haplotypes with frequencies greater than 4% were included in the analyses. Haplotypes were anchored using tag SNP genotype data and boundaries were inferred using recombination data. SLE case-control association and step-wise conditional regression data for each haplotype was generated in PLINK, as were OR (95% CI) and the association is represented by nominal uncorrected p values.

### Phenotypic association

Individuals with early age of SLE onset were classified using interquartile range and analysed using case–only format and case-control formats in SNPTest. Presence/absence of leukopenia and lymphopenia, anti-La, anti-Ro and anti-Sm autoantibody subsets, which are associated with SLE, together with renal disease, were analysed using both case-only and phenotype-control formats. Linear regression data of the most associated marker for each phenotype in each population was generated.

### B cell isolation and cell stimulation

Peripheral blood mononuclear cells (PBMC) were isolated from 40 ml whole blood from a European individual using the ACCUSPIN System-Histopaque (Sigma-Aldrich). B lymphocytes, expressers of *TNFSF4*, were negatively selected using the Dynabeads Untouched Human B Cell kit (Invitrogen). Cell purity was assessed by FACS analysis of CD19-FITC-conjugated B cells and these were 97% pure. The cells were stimulated with 25 µg/ml anti-IgM-(Fab′)_2_, 0.1 µg CD40L and 0.1 µg enhancer of CD40L to upregulate TNFSF4. Upregulation of cell-surface TNFSF4 was assessed by FACS.

### RNA isolation and 5′ rapid amplification of cDNA ends (RACE)

*T*otal RNA was isolated using the TRIzol (Sigma) method from 5×10^6^ B lymphocytes. 5′ ends of *TNFSF4* transcripts were generated by the SMARTer RACE cDNA Amplification Kit (Clontech). Primer3 was used to design gene specific primers suitable for four alternative splicing variants predicted by the Aceview alternative splicing modelling tool [34]. During PCR a universal primer was added to the 5′ end of the cDNA. In combination with each transcript specific primer, cDNA was amplified up to the 5′ end as dictated by transcript sequence and in a positive control. In order to identify clones relevant for the *TNFSF4* manuscript, we undertook colony hybridisation with a **^32^**P-labelled probe specific for the 5′ region of TNFSF4 cDNA. Following colony selection, we cloned individual PCR products using the TOPO TA Cloning Kit (Invitrogen) in order to identify individual transcript isoforms. Bacterial cultures were mini-prepped as per manufactures instructions (QIAprep Spin Miniprep Kit, Qiagen). Samples were digested with *Eco*RI and different sized fragments sequenced and Blasted against transcript sequences.

### Cis eQTL analysis

Genome-wide expression profiles stored in the Multiple Tissue Human Expression Resource (MuTHER) were available for download at http://www.muther.ac.uk/Data.html.

### Bioinformatic analysis

Transcription factors (TFs) which are predicted to interact with DNA at the risk-associated *TNFSF4* variants identified as part of this study were investigated in a sequence-specific manner. We analysed DNA-binding patterns at these locations using curated, non-redundant matrix profiles stored in the Jaspar core database [35]. In a complementary approach, putative risk loci were investigated using profiles derived from whole-genome ChIP-seq experiments on lymphoblastoid cell lines generated for the ENCODE project and stored in the Ensembl (http://www.ensembl.org/Homo_sapiens/encode.html), UCSC databases (http://genome.ucsc.edu/ENCODE/) and 1000genomes variant call format files downloaded from http://www.1000genomes.org/.

### Ethics statement

In accordance with the Department of Health's Research Governance Framework for Health and Social Care, the research project titled ‘Genetics susceptibility of systemic lupus erythematosus (SLE)’ has received favourable approval from an ethics committee and approval from the Department of Research and Development prior to commencement.

Ethics number: 06/MRE02/009

Sponsor: Wellcome Trust

Funder: King's College London

End date: 31/03/2016

R&D Approval Date: 05/08/2011

Chief Investigator: Professor Timothy Vyse

## Supporting Information

Data S1 Genomes allele frequencies for *rs1234314* and *rs2205960*.
(DOCX)

Figure S1 *Left*, Principal component (PC) 1 versus PC2 analyses of four Hapmap III African (yellow) and two Hapmap III European (red) populations and our African-American SLE-control cohort (black). *Right*. Population stratification between African-American cases (red) and controls (black) was minimised by principal components analysis using 367 major ancestry informative markers. This figure depicts the most profound ancestry differences along continuous axis of variation between cases and controls after QC filtering of the AA cohort.
(EPS)

Figure S2 PC-based analysis of the Mestizo Native American cohort (grey) and Hispanic Mestizo cohort (black) use 347 AIM SNPs.
(EPS)

Figure S3 Comparison of recombination at *TNFSF4* in African-American *TNFSF4_risk_* and *TNFSF4_non-risk_* individuals. Phased chromosomes from African-American SLE individuals homozygous for *TNFSF4*_risk_ (n = 10) and *TNFSF4*_non-risk_(n = 10) were tested for recombination using *Rhomap* from the LDHAT2.0 package. A fine-scale map of recombination rate (4Ner/kb) across 250 kb of chromosome 1q25 which encompassed *TNFSF4* and extended 5′ and 3′ regions was inferred. Individuals were identified as homozygous for *TNFSF4*_risk_ or *TNFSF4*_non-risk_. We ran *Rhomap* for a total of 1,100,000 rjMCMC iterations including a burn-in of 100000 iterations, sampling the chain after every 100. Grey diamonds indicate the location to scale of SNPs significantly associated with risk of SLE in this cohort, the *TNFSF4* gene is also located to scale under the graph.
(EPS)

Table S1 *TNFSF4* markers in African- Americans, Gullah and combined AA-Gullah (non-imputed).
(DOCX)

Table S2 (Case-only) and (Case-control) phenotype analysis.
(DOCX)
