# Supplementary material for: Trans-Ancestral Studies Fine Map the SLE-Susceptibility Locus TNFSF4
Source: PLoS Genet. 2013 Jul 18;9(7):e1003554. doi: 10.1371/journal.pgen.1003554 (PMC3715547; doi:10.1371/journal.pgen.1003554)
Supplement: Data S1 — Genomes allele frequencies for rs1234314 and rs2205960. (DOCX) [file pgen.1003554.s001.docx]

**Supplementary data**

**1000 Genomes allele frequencies, *rs2205960***

**G allele T allele**

ASW 0.9 0.1

AMR 0.71 0.29

ASN 0.77 0.23

EUR 0.79 0.21

**1000 Genomes allele frequencies, *rs1234314***

**C allele G allele**

ASW 0.64 0.36

AMR 0.54 0.46

ASN 0.62 0.38

EUR 0.58 0.42
