# Supplementary material for: Trans-Ancestral Studies Fine Map the SLE-Susceptibility Locus TNFSF4
Source: PLoS Genet. 2013 Jul 18;9(7):e1003554. doi: 10.1371/journal.pgen.1003554 (PMC3715547; doi:10.1371/journal.pgen.1003554)
Supplement: Table S1 — TNFSF4 markers in African- Americans, Gullah and combined AA-Gullah (non-imputed). (DOCX) [file pgen.1003554.s005.docx]

| **Supplementary Table 1. *TNFSF4*  markers in African- Americans, Gullah and combined AA-Gullah (non-imputed)** | | | | | | | | | | | |
| --- | --- | --- | --- | --- | --- | --- | --- | --- | --- | --- | --- |
| **Marker** | ^a.^**Location** | **A1/A2** | **African-American**  **(1529 cases, 2048 controls)** | | | **Gullah**  **(151 cases, 122 controls)** | | | **Combined (AA-Gullah)**  **(1680 cases,2170 controls)** | | |
|  |  |  | **F_A/F_U** | ***pValue*** | **ORX (95% CI)** | **F_A/F_U** | ***pValue*** | **ORX(95% CI)** | **F_A/F_U** | ***pValue*** | **ORX(95% CI)** |
| *rs7553711* | -20.96Kb 3ʹ *TNFSF4* | T/T | 0.29/0.24 | 4.01 x 10^-5^ | 1.26(1.10-1.44) | 0.23/0.16 | 0.06 | 1.51(0.98-2.34) | 0.29/0.24 | 5.34 x 10^-5^ | 1.26(1.10-1.44) |
| *rs6676785* | 7.85Kb 3ʹ *TNFSF4* | A/G | 0.29/0.24 | 3.49 x 10^-5^ | 1.26(1.10-1.44) | 0.23/0.16 | 0.06 | 1.51(0.98-2.34) | 0.20/0.24 | 6.23 x 10^-5^ | 1.26(1.1-1.44) |
| *rs10127728* | 1.72Kb 3ʹ *TNFSF4* | G/T | 0.51/0.46 | 1.30 x 10^-4^ | 1.20(1.09-1.32) | 0.46/0.39 | 0.09 | 1.34(0.95-1.89) | 0.50/0.46 | 5.68 x 10^-5^ | 1.21(1.1-1.32) |
| *rs6691738* | 0.84Kb 3ʹ *TNFSF4* | T/G | 0.29/0.25 | 6.12 x 10^-5^ | 1.25(1.09-1.43) | 0.24/0.18 | 0.11 | 1.40(0.21-0.92) | 0.29/0.25 | 6.64 x-10^-5^ | 1.25(1.09-1.44) |
| *rs3861950* | *TNFSF4* | T/C | 0.23/0.20 | 1.20 x 10^-4^ | 1.20(1.07-1.35) | 0.15/0.11 | 0.12 | 1.50(0.89-2.50) | 0.22/0.19 | 2.23 x 10^-5^ | 1.19(1.07-1.33) |
| *rs10798265* | *TNFSF4* | T/C | 0.37/0.32 | 5.17 x 10^-5^ | 0.81(0.74-0.90) | 0.42/0.4 | 0.62 | 1.09(0.77-1.54) | 0.370/0.34 | 3.05 x 10^-4^ | 0.84(0.76-0.92) |
| *rs1234314* | 0.92Kb 5' *TNFSF4* | G/C | 0.32/0.28 | 7.27 x 10^-6^ | 1.22 (1.10-1.35) | 0.34/0.24 | 0.01 | 1.62(1.10-2.37) | 0.33/0.28 | 8.84 x 10^-7^ | 1.25(1.13-1.38) |
| ***rs1234317*** | **11.3Kb 5' *TNFSF4*** | **T/G** | **0.11/0.08** | **8.15x10^-5^** | **1.34 (1.14-1.58)** | **0.09/0.05** | **6.76x10^-3^** | **2.57(1.27-5.21)** | **0.11/0.08** | **1.45 x 10^-5^** | **1.39(1.19-1.62)** |
| ***rs2205960*** | **15Kb 5' *TNFSF4*** | **T/G** | **0.07/0.04** | **8.29x10^-4^** | **1.38 (1.13-1.69)** | **0.09/0.02** | **6.51x10^-4^** | **4.69(1.78-12.38)** | **0.07/0.05** | **3.79 x 10^-5^** | **1.49(1.22-1.79)** |
| ***rs12039904*** | 35.1 Kb 5' *TNFSF4* | **T/C** | **0.06/0.04** | **3.44 x 10^-3^** | **1.36(1.11-1.69)** | **0.06/0.02** | **0.01** | **3.80(1.27-11.39)** | **0.06/0.04** | **7.60 x 10^-4^** | **1.43(1.16-1.75)** |
| *rs10912580* | 80.1Kb 5' *TNFSF4* | G/A | 0.14/0.12 | 9.24x10^-4^ | 1.25 (1.08-1.44) | 0.13/0.07 | 0.01 | 2.18(1.19-3.99) | 0.14/0.11 | 2.20 x 10^-4^ | 1.28(1.11-1.46) |
| **A1/A2- associated allele code/major allele code; F_A/F_U - allele frequency in affected/unaffected, CHISQ- chi square, ORX(95% CI)- Odds ratio (95% confidence interval).**  **After Q.C filtering African-American(1510,2022), Gullah(152,122) and both (1680,2170)**  **^a.^ Location anchored to our transcript data found by RACE-PCR and in Ensembl browser.** | | | | | | | | | | | |
