# Supplementary material for: Trans-Ancestral Studies Fine Map the SLE-Susceptibility Locus TNFSF4
Source: PLoS Genet. 2013 Jul 18;9(7):e1003554. doi: 10.1371/journal.pgen.1003554 (PMC3715547; doi:10.1371/journal.pgen.1003554)
Supplement: Table S2 — (Case-only) and (Case-control) phenotype analysis. (DOCX) [file pgen.1003554.s006.docx]

**Supplementary Table 2. (Case-only) and (Case-control) phenotype analysis**

|  | | **European** | | | | **Hispanic** | | | | **AA + Gullah** | | |
| --- | --- | --- | --- | --- | --- | --- | --- | --- | --- | --- | --- | --- |
|  | Presence/  Absence | | Best  Marker | *P_U_*/ OR (95%CI) | Presence/  Absence | | Best  Marker | *P_U_*/ OR (95%CI) | Presence/  Absence | | Best  Marker | *P_U_*/ OR (95%CI) |
|  |  |  |  |  |  |  |  |  |  |  |  |  |
| ***Phenotype-case*** |  | |  |  |  | |  |  |  | |  |  |
|  |  | |  |  |  | |  |  |  | |  |  |
| Age of onset, median (IQR) | 816/774 | | rs12405577 | 1.43x10^-3^/0.78 (0.68-0.91) | 139/138 | | rs1539259 | 1.44x10^-3^/ 0.57(0.41-0.81) | 337/ 269 | | RS844654 | 2.51 x 10^-3^/0.70(0.5560.89) |
| Anti-dsDNA | 1177/1904 | | rs12124768 | 3.5x10^-5^/0.74(0.6-0.9) | 515/522 | | rs12405577 | 3.8x10^-4^/ 1.4(1.16-1.68) | 752/830 | | RS4250 | 3.53 x 10^-3^/2.42(1.31-4.46) |
| Anti-Ro | **422/1742** | | **rs1234317** | **9.5x10^-4^/1.31 (1.12-1.54)** | **119/385** | |  |  | 330/648 | | RS10127727 | 5.98x 10^-3^/0.74(0.59-0.92) |
| Anti-Sm | **225/2461** | | **rs2205960** | **0.05/1.23(1-1.52)** | 204/772 | | rs12405577 | 3.1x10^-3^/1.41(1.12-1.78) | **487/763** | | **rs2205960** | **5.1x10^-3^/1.57(1.14-2.16)** |
| Renal Disease | **1054/2020** | | **rs2205960** | **2.87 x 10^-4^/1.24 (1.1-1.4)** | 439/449 | | rs16845607 | 0.03/1.3(1.02-1.77) | 785, 784 | | RS7518045 | 1.77 x 10^-3^/0.73(0.60-0.89) |
| Immunologic | 2441/570 | | rs1234313 | 2.5 x 10^-3^/0.81(0.7-0.93) | 297/92 | | rs13343108 | 1.7x10^-4^/0.52(0.36-0.73) | **1229/167** | | **RS2205960** | **0.035/1.77(1.04-3.04)** |
|  |  | |  |  |  | |  |  |  | |  |  |
|  |  | |  |  |  | |  |  |  | |  |  |
|  |  | |  |  |  | |  |  |  | |  |  |
| ***Phenotype-control*** | | | | | | | | | | | | |
|  |  | |  |  |  | |  |  |  | |  |  |
| Age of onset, median (IQR) | 816/3580 | | rs1234317 | 6.7x10^-4^/1.3(1.23-1.52) | 139/615 | | rs2205960 | 1.89x10^-3^/ 1.55(1.17-2.03) | 337/2144 | | RS10489265 | 4.57 x 10^-4^/1.39(1.11-1.74) |
| Anti-dsDNA | 1177/3580 | | rs2205960 | 6.5x10^-9^/1.37(1.23-1.52) | 515/615 | | rs12039904 | 6.12x10^-10^/ 1.74(1.46-2.07) | 752/2144 | | RS2205960 | 1.59 x 10^-6^/1.74(1.39-2.19) |
| Anti-Ro | **422/3580** | | **rs1234317** | **9.5x10^-8^/1.52 (1.3-1.76)** | **119/615** | |  |  | 330/2144 | | RS1234314 | 1.67 x 10^-3^/1.32(1.11-1.58) |
| Anti-Sm | **225/3580** | | **rs2205960** | **1.63x10^-5^ /1.58(128-1.94)** | 204/615 | | rs2205960 | 2.4x10^-7^/1.85(1.46-2.4) | **487/2144** | | **RS2205960** | **6.67 x 10^-7^/1.91(1.47-2.47)** |
| Renal Disease | **1054/3580** | | **rs2205960** | **2.87x10^-14^ /1.53 (1.37-1.70)** | 439/615 | | rs2205960 | 3.24x10^-9^/1.75(1.45-2.1) | 785, 2170 | | RS1234317 | 1.08 x 10^-5^/1.52(1.26-1.83) |
| Immunologic | 2441/3575 | | rs2205960 | 2.2x10^-15^/1.4(1.29-1.53) | 783/615 | | rs2205960 | 2.57x10^-10^/1.69(1.43-1.98) | **1229, 2170** | | **RS2205960** | **5.42 x 10^-6^/1.59(1.30-1.95)** |
|  |  | |  |  |  | |  |  |  | |  |  |
|  |  | |  |  |  | |  |  |  | |  |  |
| **Case and control numbers after filtering for QC. Bold Indicates same marker is most-associated with phenotype in case-only and case-control analyses** | | | | | | | | | | | | |
